# Burden and trends of cancer attributable to occupational asbestos exposure in China from 1990 to 2021

**DOI:** 10.3389/fpubh.2025.1672598

**Published:** 2026-01-05

**Authors:** Bijuan Chen, Xiuhui Zheng, Hanchen Zheng, Hongju Chen, Shaohua Xu, Qifei Li, Hui Lin, Jiami Yu, Yun Xu, Zengqing Guo, Zhouwei Zhan

**Affiliations:** 1 Department of Radiation Oncology, Clinical Oncology School of Fujian Medical University, Fujian Cancer Hospital, Fuzhou, Fujian, China; 2 Department of Medical Affairs, Clinical Oncology School of Fujian Medical University, Fujian Cancer Hospital, Fuzhou, Fujian, China; 3 Department of Medical Oncology, Clinical Oncology School of Fujian Medical University, Fujian Cancer Hospital, Fuzhou, Fujian, China; 4 Department of Gynecology, Clinical Oncology School of Fujian Medical University, Fujian Cancer Hospital, Fuzhou, Fujian, China; 5 Department of Hepatobiliary and Pancreatic Surgery, Clinical Oncology School of Fujian Medical University, Fujian Cancer Hospital, Fuzhou, Fujian, China

**Keywords:** occupational asbestos exposure, cancer burden, DALYs, joinpoint analysis, China, global burden of disease

## Abstract

**Background:**

Occupational exposure to asbestos remains a major public health concern in China, particularly due to the long latency of asbestos-related diseases and the persistent use of asbestos in male-dominated industries. However, the long-term trends and drivers of asbestos-related cancer burden have not been fully quantified.

**Methods:**

This study used data from the Global Burden of Disease Study 2021 to estimate the burden of total cancer attributable to occupational asbestos exposure in China from 1990 to 2021. Key indicators included deaths, disability-adjusted life years (DALYs), years lived with disability (YLDs), and years of life lost (YLLs), disaggregated by age, sex, cancer type, and time period. Joinpoint regression and age-period-cohort analyses were conducted to explore temporal dynamics, while decomposition analysis assessed the contributions of population growth, aging, and epidemiological changes.

**Results:**

In 2021, occupational asbestos exposure led to 29,020 deaths and 535,732 DALYs in China, with a significantly higher burden among males. The number of cases rose steadily from 1990, with sharp increases between 2000 and 2010, plateauing thereafter and rising again after 2017. Age-standardized rates showed dynamic trends: increasing until 2010–2011, declining until 2018, and rebounding modestly, particularly among females. The burden was highest among individuals aged 85–94 and showed a rightward shift over time. Lung cancer accounted for over 85% of asbestos-related DALYs, followed by mesothelioma, ovarian, and laryngeal cancers. Joinpoint and age-period-cohort analyses highlighted long-latency effects and generational shifts, while decomposition showed that aging and epidemiological changes were the dominant drivers of increased deaths, whereas DALYs were primarily driven by epidemiological factors and population growth.

**Conclusion:**

The burden of asbestos-related cancers in China remains substantial and is rising due to demographic aging and persistent exposure risks. Stronger occupational safety regulations, exposure surveillance, and asbestos bans are urgently needed to curb future disease burden.

## Introduction

Asbestos is a naturally occurring fibrous silicate mineral long valued for its heat resistance and durability in construction, shipbuilding, and insulation. However, extensive evidence shows that exposure to asbestos fibers, especially in occupational settings, causes several cancers, including lung cancer, mesothelioma, laryngeal cancer, and ovarian cancer (1–3). The International Agency for Research on Cancer (IARC) classifies all forms of asbestos as Group 1 human carcinogens (4, 5). Despite this, chrysotile asbestos remains widely used in some low- and middle-income countries, including China. As the world’s second-largest asbestos producer and leading consumer, China faces a major public health challenge from both historical and ongoing asbestos use, particularly in male-dominated industries such as construction and manufacturing (6–8). Although more than 60 countries have banned asbestos, China has yet to adopt a comprehensive national ban, and the long-term disease burden from asbestos exposure continues to rise.

The health impact of asbestos is complex and long lasting, largely because asbestos-related diseases have long latency periods that range from less than 10 years for asbestosis, to about 20–40 years for lung cancer, and often more than 40–60 years for mesothelioma after initial exposure (9, 10). The Global Burden of Disease (GBD) Study provides a framework for estimating population level cancer burden attributable to specific occupational exposures, including asbestos, using metrics such as deaths, disability adjusted life years (DALYs), years lived with disability (YLDs), and years of life lost (YLLs) (11–13). In China, the burden of asbestos-related cancers remains underreported and underestimated, particularly among older men exposed many years ago. Trends in disease burden are shaped not only by exposure levels but also by demographic changes, such as population aging and growth, and by advances in cancer detection and treatment. Previous studies suggest that mesothelioma, once considered rare in China, has increased markedly, while lung cancer continues to dominate the asbestos-attributable cancer profile (14, 15). However, a comprehensive national analysis of the long term cancer burden due to occupational asbestos exposure in China has been lacking.

This study aims to quantify the burden and temporal trends of total cancer attributable to occupational asbestos exposure in China from 1990 to 2021 using GBD 2021 data, stratified by age and sex, to inform prevention and policy. By concisely defining the scope and objectives, we provide an evidence base to support worker protection, enhanced occupational health surveillance, and progress toward a national asbestos ban.

## Methods

### Data source

This study used data from the Global Burden of Disease (GBD) Study 2021, developed by the Institute for Health Metrics and Evaluation. The GBD framework provides comprehensive and comparable estimates of disease burden attributable to many risk factors, including occupational asbestos exposure, across 204 countries and territories (11, 12). For this analysis, we extracted data for China from 1990 to 2021, including estimates of deaths, DALYs, YLDs, and YLLs attributable to occupational asbestos exposure, both for total cancers and for specific cancer sites. Age-, sex-, and cause-specific estimates were obtained from the GBD Results Tool and visualization platforms, which integrate national disease registries, occupational exposure data, vital registration systems, and population censuses. Asbestos exposure in the GBD is modeled using exposure prevalence, relative risks from meta-analyses, and population-attributable fractions (PAF). Estimates are generated using statistical tools such as the Cause of Death Ensemble Model (CODEm) and DisMod-MR 2.1 to ensure consistency across epidemiological measures (16). All estimates include 95% uncertainty intervals based on 1,000 posterior draws to account for uncertainty in data inputs, model structure, and parameter estimation. The GBD methodology has been validated for assessing occupational cancer burden globally and provides a standardized approach for long-term burden analysis (8).

### Definition and estimation

In the GBD Study 2021, occupational asbestos exposure is defined using the Asbestos Impact Ratio (AIR), which serves as a proxy for population level exposure. The AIR is calculated as the ratio of excess mesothelioma deaths in the general population to excess mesothelioma deaths in a reference population with high occupational exposure (11, 16). This method uses the strong and specific causal relationship between asbestos and mesothelioma to estimate exposure response patterns across countries and over time. The resulting population attributable fractions are then applied to total cancer burden to estimate cancer cases and deaths attributable to occupational asbestos exposure. In this analysis, total cancer refers to all malignant neoplasms classified under ICD-10 codes C00 to C97 and excludes benign and *in situ* neoplasms, along with neoplasms of uncertain behavior, such as those under ICD-10 codes D00 to D48 (11). Included cancers span the trachea, bronchus, lung, mesothelium, larynx, ovary, and other organs, as well as non-melanoma skin cancers. Estimates of deaths, DALYs, YLDs, and YLLs attributable to asbestos exposure were calculated by multiplying disease burden by the respective population attributable fractions for each cancer site, sex, age group, and year. All estimates include 95 percent uncertainty intervals derived from 1,000 posterior draws.

### Descriptive analysis

Descriptive statistical analyses were conducted to assess the burden and distribution of total cancer attributable to occupational asbestos exposure in China in 2021 and across the period from 1990 to 2021. We evaluated deaths, DALYs, YLDs, and YLLs stratified by sex and five-year age groups. Age standardized rates per 100,000 population were calculated using the GBD global standard population to allow temporal comparison independent of demographic change (11, 16, 17). To characterize age and sex patterns in 2021, absolute numbers and age standardized rates were plotted across age groups for males and females. Crude numbers and rates were also compared between 1990 and 2021 to illustrate changes in burden by age. Cause specific estimates were disaggregated to identify leading contributors, with particular attention to cancers of the trachea, bronchus, and lung, mesothelioma, ovary, and larynx. Their proportional contributions to total deaths and DALYs were examined. Line graphs, bar charts, and stacked plots were used to visualize age specific and temporal patterns, and sex specific differences were emphasized to highlight the disproportionate burden among men due to historically higher occupational exposure. All results include 95 percent uncertainty intervals to reflect variability in the modeled estimates.

### Joinpoint regression analysis

Joinpoint regression analysis was used to quantify temporal trends in the age standardized rates of cancer burden attributable to occupational asbestos exposure in China from 1990 to 2021. This method identifies statistically significant changes in trend, known as joinpoints, and calculates the annual percentage change (APC) for each segment and the average annual percentage change (AAPC) across the entire study period. Analyses were conducted separately for age standardized mortality, DALY, YLD, and YLL rates. Sex stratified models were also constructed to assess differences between males and females. Modeling was performed using the joinpoint Regression Program version 5.2.0 developed by the United States National Cancer Institute. A log linear model was applied, and the number of joinpoints was selected using a permutation test with a maximum of five joinpoints to allow for detection of nonlinear trends (18). Statistical significance was set at 0.05. Annual percentage change and average annual percentage change values were reported with 95 percent confidence intervals to reflect uncertainty in the trend estimates.

### Age-period-cohort (APC) analysis

To examine the independent effects of age, period, and birth cohort on asbestos-related cancer burden in China, an APC model was applied. This approach separates age-related biological risk, period-related environmental or policy influences, and cohort-specific differences shaped by historical patterns of asbestos exposure. Because age, period, and cohort are linearly dependent, the intrinsic estimator (IE) method was used to obtain unbiased effect estimates (19, 20). DALY and mortality data for total cancer attributable to occupational asbestos exposure from 1990 to 2021 were taken from the GBD 2021 database and organized into consecutive five-year age groups (20–24 to 90–94 years), five-year periods (1992–1996 to 2017–2021), and corresponding birth cohorts. Individuals younger than 20 years and older than 95 years were excluded to maintain model stability. Age categories followed standard epidemiological groupings, and period classifications were aligned accordingly. Birth cohort values were derived by subtracting age midpoints from period midpoints. The analysis was conducted using the Epi package (version 2.46) in R (version 4.3.1). Model adequacy was evaluated by examining residuals and comparing Akaike Information Criterion values across alternative model specifications.

### Decomposition analysis

To quantify the contributions of demographic and epidemiologic factors to changes in asbestos-related cancer burden over time, a decomposition analysis was conducted. This approach separates total changes in disease burden into three components: population growth, population aging, and epidemiologic change. Epidemiologic change refers to variation in age-specific cancer burden not explained by demographic shifts and may reflect changes in exposure prevalence, case detection, diagnostic capacity, or treatment outcomes (21, 22). The analysis examined changes in the absolute number of deaths and DALYs attributable to occupational asbestos exposure in China between 1990 and 2021. A stepwise replacement algorithm was applied, in which each factor was introduced sequentially while holding the others constant. Age-specific death and DALY rates, population structure, and total population size were taken from the GBD 2021 database. Analyses were stratified by sex to assess gender-specific contributions.

## Results

### Burden of total cancer attributable to occupational asbestos exposure in China, 2021

In 2021, occupational asbestos exposure continued to cause a substantial cancer burden in China (Table 1). An estimated 29,020 cancer deaths were attributable to asbestos exposure, with males accounting for most of the cases. This corresponded to an age standardized death rate of 1.51 per 100,000 population, with a much higher rate in males (2.67) than in females (0.71). The total disease burden reached 535,732 DALYs, reflecting both premature mortality and morbidity. Males experienced more than twice the DALYs of females, consistent with historically higher exposure in male dominated industries. The age standardized DALY rate was 25.88 per 100,000, again showing a large sex difference (42.74 for males versus 12.73 for females). Years of life lost made up the vast majority of DALYs, indicating that premature death remains the primary contributor to the asbestos-related cancer burden, while years lived with disability accounted for a smaller share. Even so, the disability burden still represents a meaningful reduction in quality of life for surviving patients.

**Table 1:** All-age cases and age-standardized deaths, DALYs, YLDs, and YLLs rates in 2021 for total cancer attributable to occupational asbestos exposure in China.

Measure	All-ages cases			Age-standardized rates per 100,000 people		
	Total	Male	Female	Total	Male	Female
Deaths	29,020 (18,442, 42,779)	21,329 (12,696, 33,520)	7,691 (4,203, 12,250)	1.51 (0.96, 2.21)	2.67 (1.59, 4.15)	0.71 (0.39, 1.13)
DALYs	535,732 (346,751, 802,494)	393,608 (232,825, 631,854)	142,124 (78,859, 224,740)	25.88 (16.70, 38.43)	42.74 (25.50, 67.28)	12.73 (7.05, 20.20)
YLDs	7,071 (4,146, 11,227)	5,131 (2,735, 8,516)	1940 (1,030, 3,268)	0.35 (0.20, 0.55)	0.59 (0.31, 0.96)	0.17 (0.09, 0.29)
YLLs	528,661 (342,220, 792,787)	388,477 (229,503, 623,493)	140,184 (77,795, 221,769)	25.53 (16.52, 37.91)	42.15 (25.14, 66.35)	12.56 (6.95, 19.93)

Values in parentheses indicate 95% UIs, estimated using Monte Carlo simulations. DALYs, disability-adjusted life-years; YLDs, years lived with disability; YLLs, years of life lost; UI, uncertainty interval.

### Age and sex patterns of cancer burden attributable to occupational asbestos exposure in China, 2021

**Figure 1:**
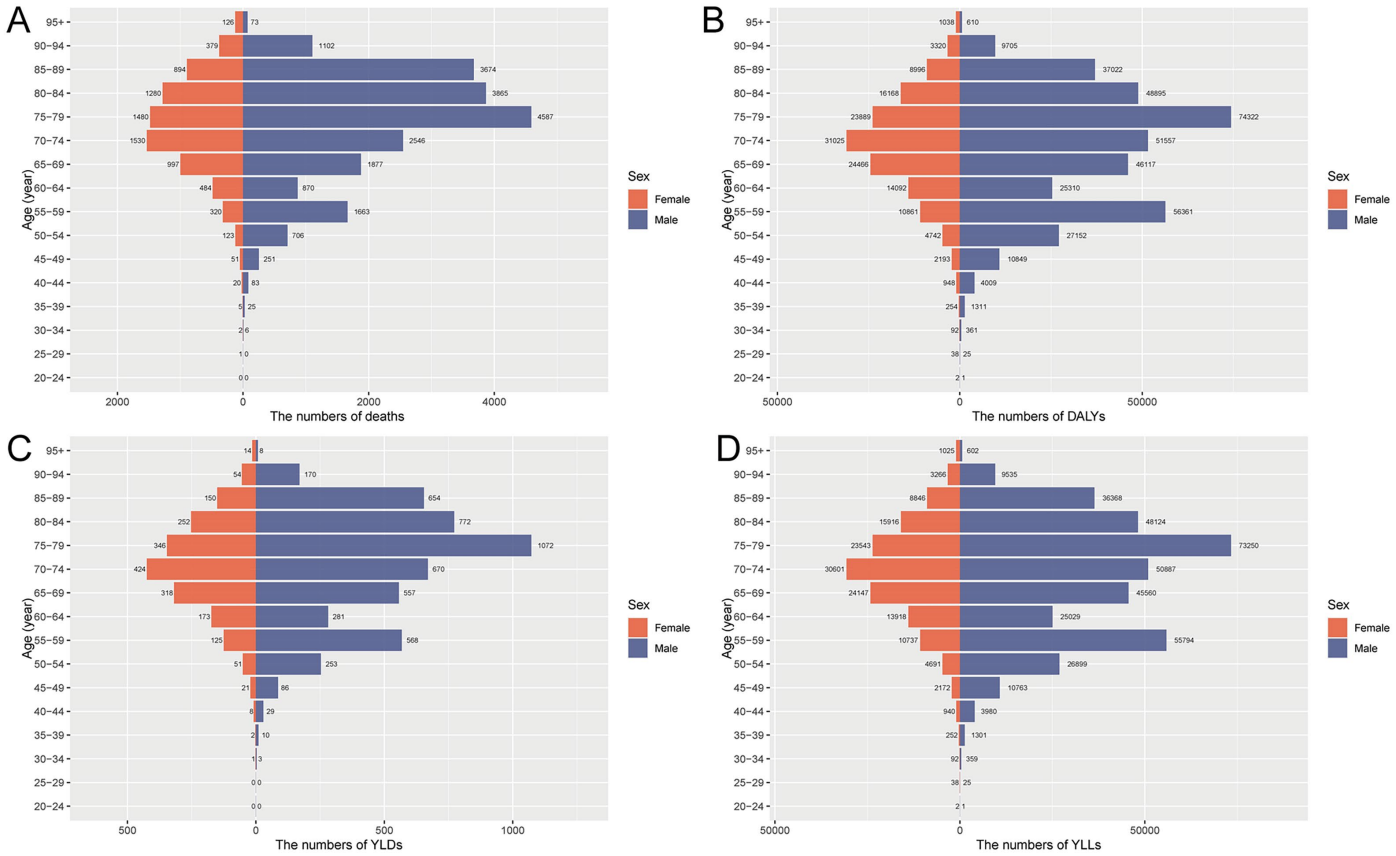
Age- and sex-specific disease burden of total cancer attributable to occupational asbestos exposure in China, 2021. **(A)** Number of deaths by age group and sex. **(B)** Number of DALYs by age group and sex. **(C)** Number of YLDs by age group and sex. **(D)** Number of YLLs by age group and sex. DALYs, disability-adjusted life years; YLDs, years lived with disability; YLLs, years of life lost.

**Figure 2:**
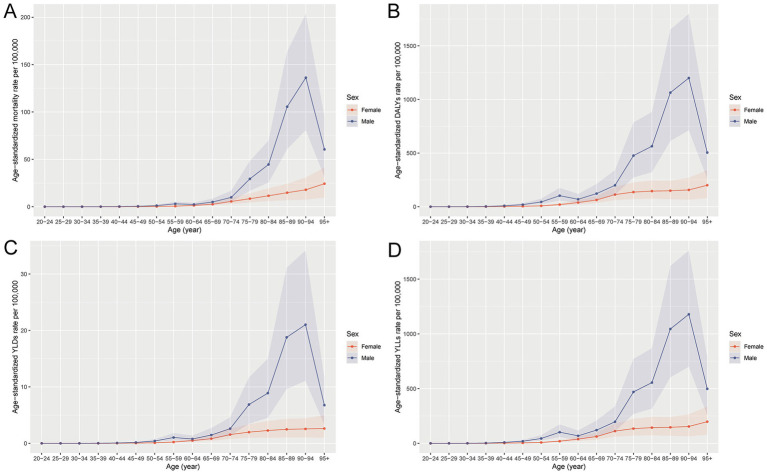
Age-specific trends in age-standardized rates of total cancer attributable to occupational asbestos exposure in China, 2021. **(A)** Age-standardized mortality rate per 100,000 population by age group and sex. **(B)** Age-standardized DALY rate per 100,000 population by age group and sex. **(C)** Age-standardized YLD rate per 100,000 population by age group and sex. **(D)** Age-standardized YLL rate per 100,000 population by age group and sex. DALYs, disability-adjusted life years; YLDs, years lived with disability; YLLs, years of life lost.

In 2021, the burden of total cancer attributable to occupational asbestos exposure in China showed marked differences by age and sex. Absolute numbers of deaths, DALYs, YLDs, and YLLs increased steadily with age and were consistently higher in males, reflecting the concentration of asbestos exposure in male-dominated industries. A bimodal pattern appeared across most indicators, with peaks in the 55–59 and 75–79 age groups, the latter being more prominent. This pattern points to both the long-term legacy of historical exposure and the cumulative impact in older adults. YLLs accounted for most DALYs at all ages, underscoring that premature mortality remains the main contributor to asbestos-related cancer burden. Although YLDs were relatively low, they increased in middle-aged and older groups, indicating a growing disability burden among survivors. Age-standardized rates for deaths, DALYs, and YLLs rose sharply after age 65 and reached their highest levels among individuals aged 85–94, mirroring the trends observed in absolute numbers (Figures 1, 2).

### Temporal trends and age-specific changes in cancer burden attributable to occupational asbestos exposure from 1990 to 2021

**Figure 3:**
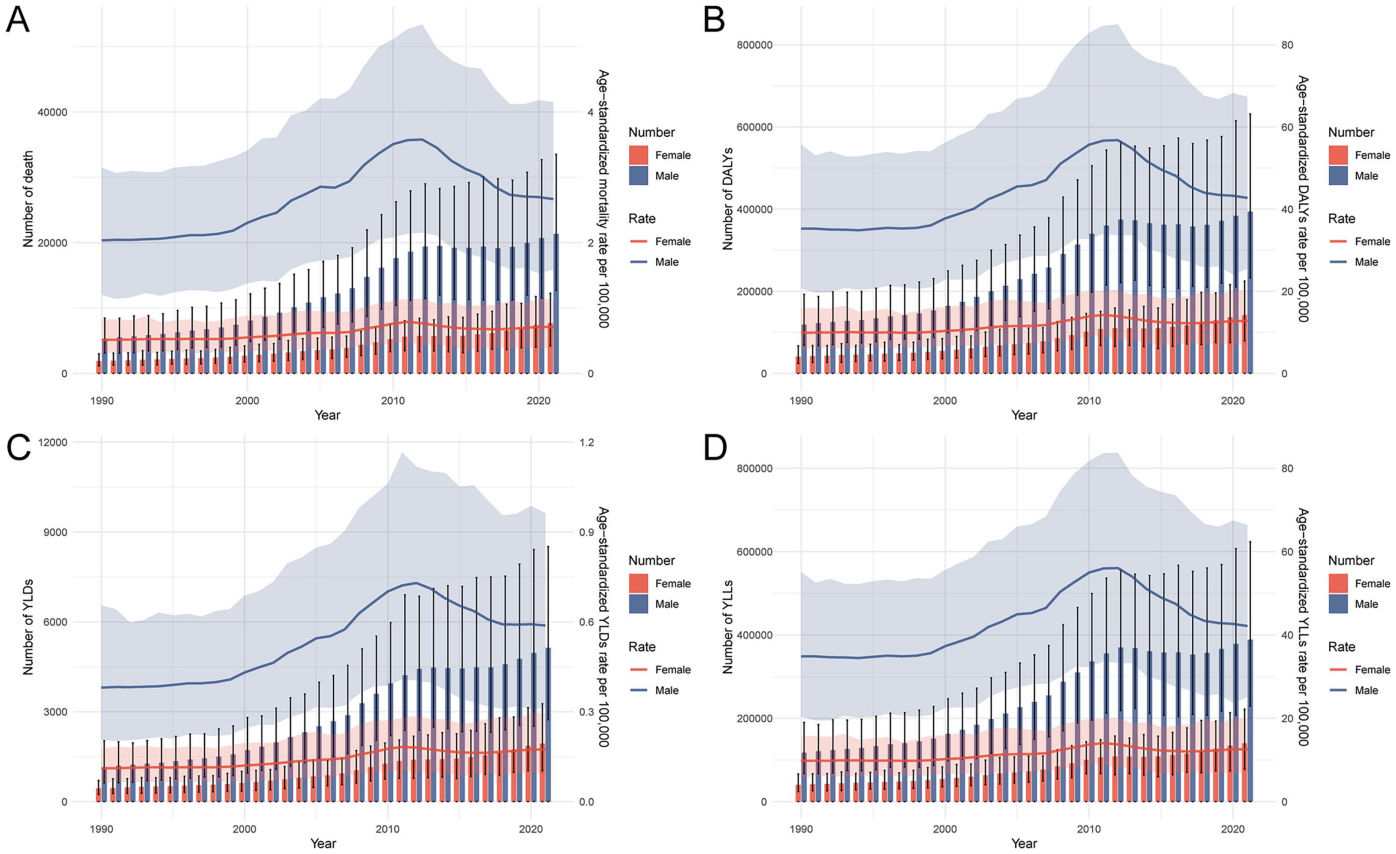
Trends in number and age-standardized rates per 100,000 population of total cancer burden attributable to occupational asbestos exposure in China, 1990–2021. **(A)** Number and age-standardized rate of deaths by sex. **(B)** Number and age-standardized rate of DALYs by sex. **(C)** Number and age-standardized rate of YLDs by sex. **(D)** Number and age-standardized rate of YLLs by sex. DALYs, disability-adjusted life years; YLDs, years lived with disability; YLLs, years of life lost.

From 1990 to 2021, both the number and age-standardized rates of deaths, DALYs, YLDs, and YLLs for total cancer attributable to occupational asbestos exposure in China demonstrated evolving and sex-specific patterns (Figure 3, Supplementary Figure S1). The absolute number of cases rose steadily. There was a sharp increase from 2000 to 2010, a leveling off in the early 2010s, and a renewed rise since 2017, particularly among men. In contrast, age-standardized rates showed dynamic trends: rising significantly until 2010–2011, declining thereafter, and stabilizing or rebounding modestly in recent years, particularly among females. Age-specific analysis further revealed that, in 2021, the number of asbestos-related cancer cases was markedly higher than in 1990 across all age groups, reflecting the compounding effects of population growth, aging, and latency. Furthermore, in 2021, the age-specific rates started to surpass those of 1990 beginning from the age group of 70–74. The most significant differences were noted in the 85–94 age range, and in both years, this age group also exhibited the highest burden.

### Site-specific and sex-specific patterns of cancer burden attributable to occupational asbestos exposure in China, 2021

**Figure 4:**
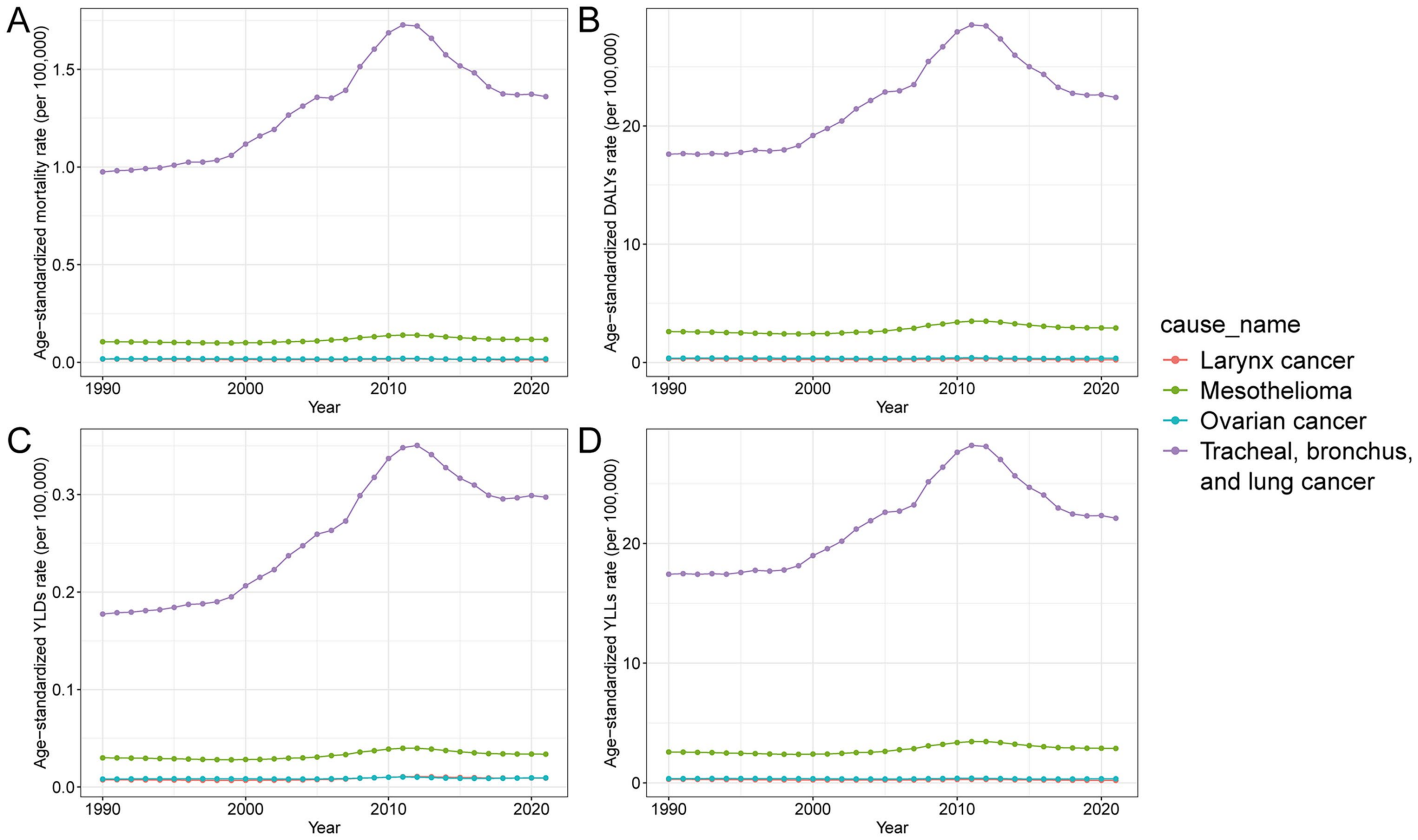
Trends in age-standardized rates of cancer burden attributable to occupational asbestos exposure in China by cause, 1990–2021. **(A)** Age-standardized mortality rate per 100,000 population by cancer type. **(B)** Age-standardized DALY rate per 100,000 population by cancer type. **(C)** Age-standardized YLD rate per 100,000 population by cancer type. **(D)** Age-standardized YLL rate per 100,000 population by cancer type. DALYs, disability-adjusted life years; YLDs, years lived with disability; YLLs, years of life lost.

In 2021, cancers of the trachea, bronchus, and lung accounted for most of the cancer burden attributable to occupational asbestos exposure in China, contributing 89.7 percent of total deaths and 86.0 percent of DALYs, followed by mesothelioma, ovarian cancer, and laryngeal cancer (Table 2). The age standardized mortality and DALY rates for lung cancer were 1.4 and 22.4 per 100,000, respectively, and both increased compared with 1990. Mesothelioma and laryngeal cancers contributed smaller burdens and showed relatively stable or declining age standardized trends. YLLs made up most of the burden for all cancer types, while YLDs, although lower, increased modestly, especially for lung cancer, which experienced a 67.6 percent rise in its age standardized YLD rate since 1990 (Supplementary Table S1). Temporal trends indicated that lung cancer remained the primary driver of the overall increase in burden over time (Figure 4). Males bore most of the burden across all indicators and cancer types, particularly for lung cancer and mesothelioma. Ovarian cancer showed a burden exclusively in females, and laryngeal cancer also displayed strong male predominance (Supplementary Figure S2).

**Table 2:** Mortality and DALYs for total cancer attributable to occupational asbestos exposure in China, 2021, with trends in ASRs per 100,000 population, 1990–2021.

Cancer type	Deaths			DALYs		
	No. in thousands	Age-standardized rate per 100,000	Percentage change from 1990 to 2021	No. in thousands	Age-standardized rate per 100,000	Percentage change from 1990 to 2021
Total cancers	29 (18.4, 42.8)	1.5 (1, 2.2)	35.3 (−1.8, 83.1)	535.7 (346.8, 802.5)	25.9 (16.7, 38.4)	23.9 (−12.2, 74.7)
Tracheal, bronchus, and lung cancer	26 (15.8, 39.3)	1.4 (0.8, 2.1)	39.6 (0.1, 92.6)	460.9 (279.1, 712.2)	22.4 (13.5, 34.4)	27.2 (−11.3, 84.1)
Mesothelioma	2.4 (1.9, 3.1)	0.1 (0.1, 0.1)	11.2 (−22, 48.7)	62.8 (46.5, 81.3)	2.9 (2.1, 3.8)	11.6 (−26.2, 58.1)
Ovarian cancer	0.4 (0.1, 0.7)	0 (0, 0)	−1.6 (−43.8, 62.1)	7.6 (2.6, 14.7)	0.3 (0.1, 0.7)	−3.2 (−47, 66.6)
Larynx cancer	0.2 (0.1, 0.4)	0 (0, 0)	−25.3 (−47.6, 6)	4.4 (2.3, 7.3)	0.2 (0.1, 0.4)	−29.4 (−53, 6.9)

Values in parentheses indicate 95% UIs, estimated using Monte Carlo simulations. DALYs, disability-adjusted life years; ASRs, age-standardized rates; UI, uncertainty interval.

### Temporal trends by sex in age-standardized rates of asbestos-related cancer burden based on joinpoint regression analysis

**Figure 5:**
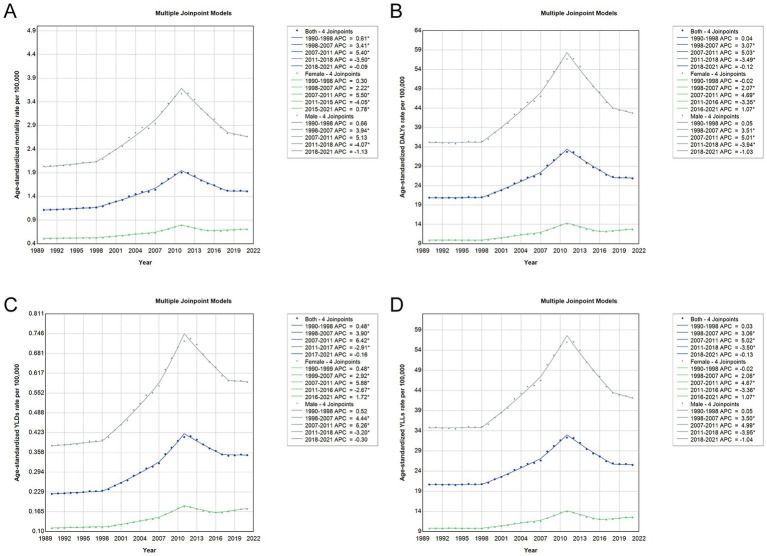
Joinpoint regression analysis of age-standardized rates for total cancer attributable to occupational asbestos exposure in China by sex, 1990–2021. **(A)** Age-standardized mortality rate (ASMR). **(B)** Age-standardized DALY rate. **(C)** Age-standardized YLD rate. **(D)** Age-standardized YLL rate. Blue lines represent trends in the total population, green lines represent females, and grey lines represent males. APC, annual percentage change; DALY, disability-adjusted life year; YLD, years lived with disability; YLL, years of life lost.

Between 1990 and 2021, age standardized rates of asbestos-related cancer burden in China showed marked temporal variation across sexes and time periods (Supplementary Table S2, Figure 5). Joinpoint regression revealed a steep rise in age standardized mortality, DALY, YLL, and YLD rates from 1998 to 2011, with annual percent changes greater than 3 percent for all indicators in both sexes. The increase was most pronounced between 2007 and 2011, particularly for DALYs and YLLs, where annual percent changes exceeded 5 percent. After this peak, all indicators declined significantly during 2011 to 2018, suggesting possible effects of industrial restructuring or reduced exposure. From 2018 onward, trends leveled off or showed slight increases, most notably among females, who experienced renewed rises in age standardized DALY rates (average annual percent change 1.07, 95 percent confidence interval 0.50 to 1.87) and YLD rates (average annual percent change 1.72, 95 percent confidence interval 1.18 to 2.54). Across the entire period, males consistently had higher age standardized rates than females for all indicators, reflecting the long-standing concentration of asbestos exposure in male-dominated industries.

### Age-period-cohort (APC) effects on DALY and mortality rates of asbestos-related cancer in China

**Figure 6:**
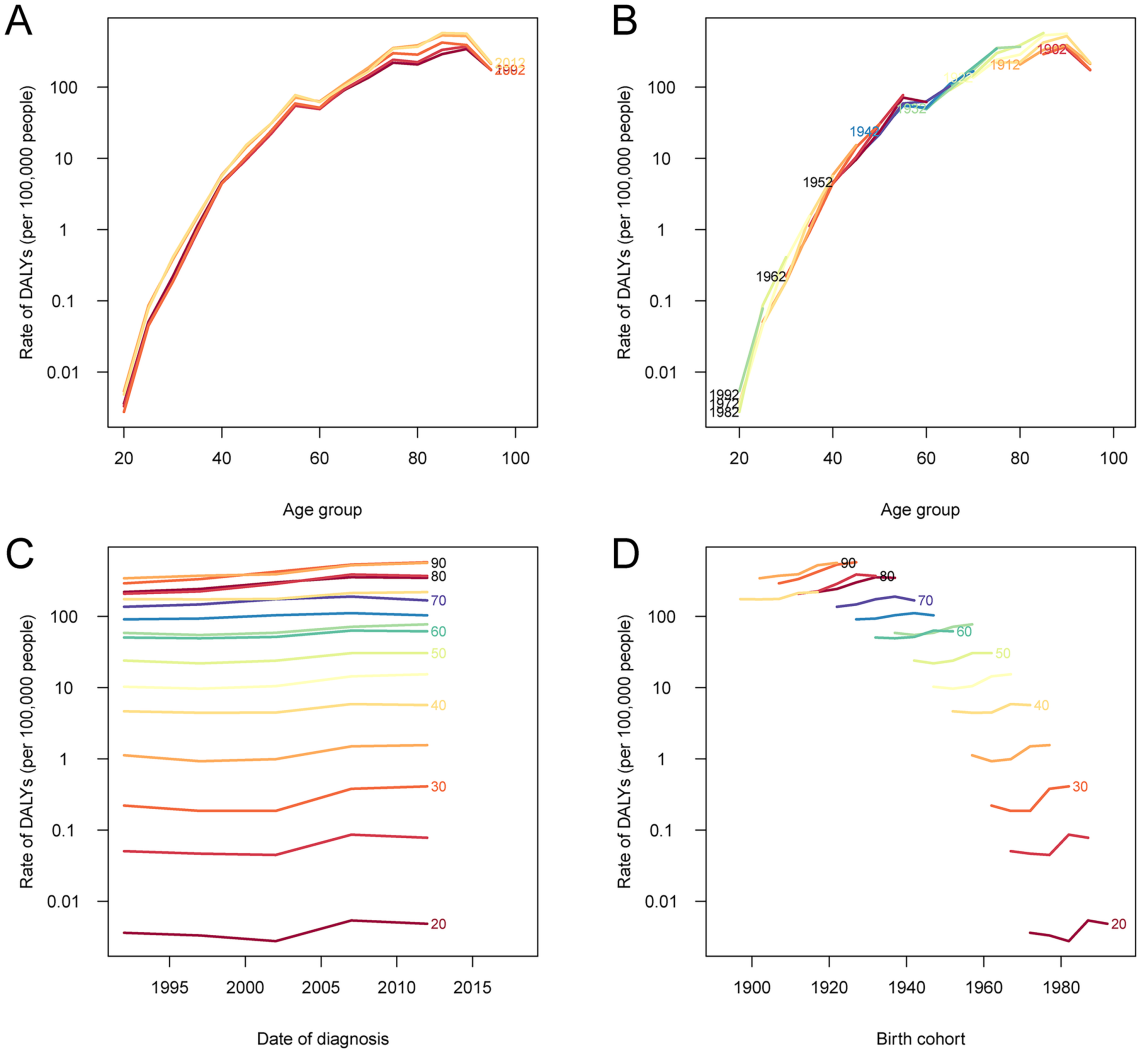
Age-period-cohort analysis of DALY rates for total cancer attributable to occupational asbestos exposure in China, 1990–2021. **(A)** The age-specific DALY rates according to time periods; each line connects the DALY rates for a given 5-year period. **(B)** The age-specific DALY rates according to birth cohorts; each line connects the DALY rates for a given 5-year birth cohort. **(C)** The period-specific DALY rates according to age groups; each line connects the DALY rates for a given 5-year age group. **(D)** The cohort-specific DALY rates according to age groups; each line connects the DALY rates for a given 5-year age group. DALY, disability-adjusted life year.

The APC analysis showed clear temporal and generational patterns in the DALY and death rates associated with total cancer attributable to occupational asbestos exposure in China from 1990 to 2021 (Figure 6, Supplementary Figure S3). Age-specific rates for both indicators rose steadily with advancing age and increased sharply after 60 years, peaking in the 85–89 age group, a pattern consistent across all time periods. Period effects indicated a general rise from 1990 to 2010 across most ages, followed by a plateau or slight decline after 2010, particularly among younger age groups. Cohort patterns revealed progressively lower DALY and death rates in later birth cohorts born after 1940 at comparable ages, suggesting improvements in workplace safety or reductions in asbestos exposure in more recent decades. In contrast, older cohorts, particularly those born between 1900 and 1935, showed markedly higher rates, especially beyond age 70, reflecting the cumulative impact of long-term occupational exposure over their lifetimes.

### Drivers of change in asbestos-related cancer burden in China from 1990 to 2021

**Figure 7:**
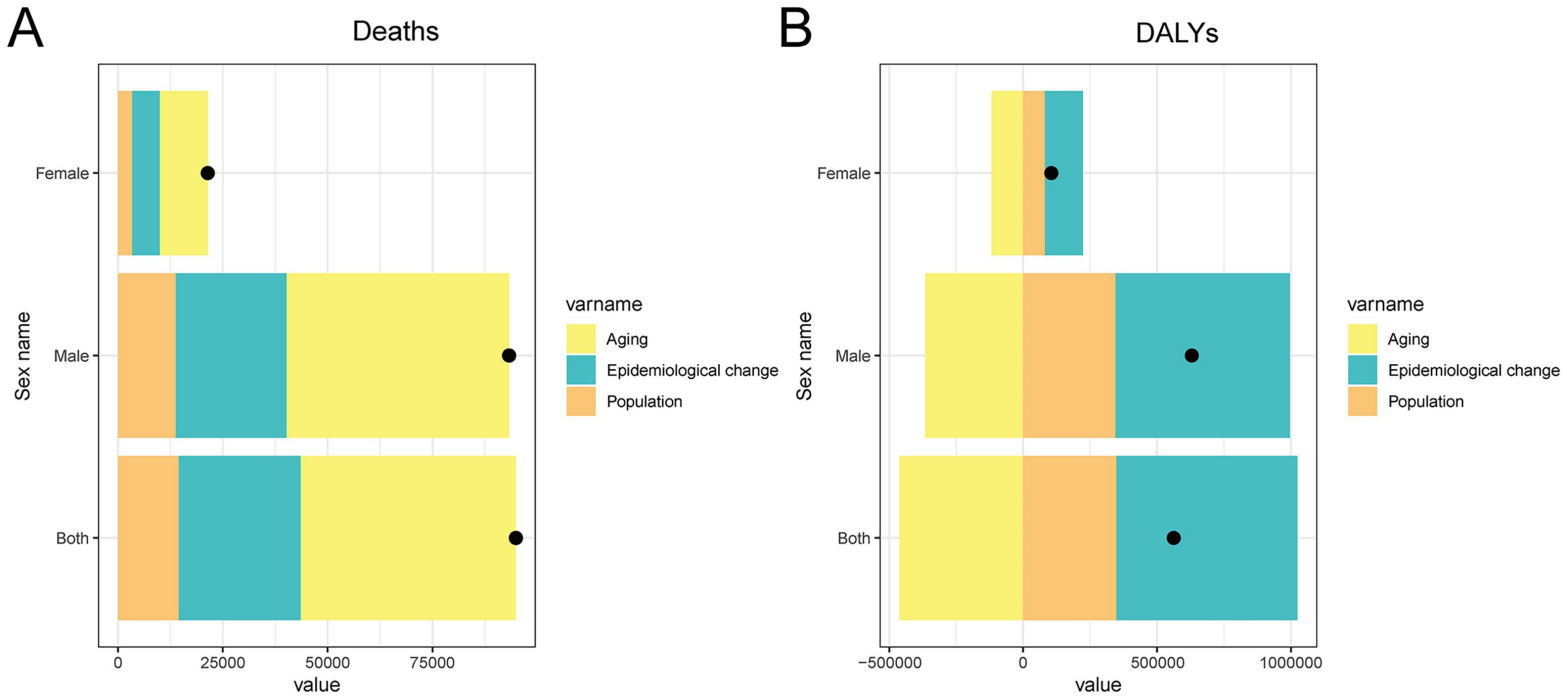
Decomposition of changes in deaths **(A)** and DALYs **(B)** attributable to total cancer caused by occupational asbestos exposure in China from 1990 to 2021. The contributions of three main components are shown: population growth, population aging, and epidemiological change, stratified by sex (both sexes, male, and female). Bars represent the absolute change in number of deaths and DALYs attributable to each factor. DALY, disability-adjusted life year.

The decomposition analysis showed clear and differing contributions of demographic and epidemiologic factors to the rise in total cancer deaths and DALYs attributable to occupational asbestos exposure in China from 1990 to 2021 (Figure 7). For deaths, population aging was the largest contributor, followed by epidemiologic change, with population growth playing a smaller role. For DALYs, epidemiologic change was the dominant driver, followed by population growth, while aging had a mitigating effect, likely because older individuals experience fewer years of productivity loss and fewer years lived with disability. All three components were consistently larger in males than in females, reflecting men’s historically higher occupational exposure to asbestos. These findings highlight the complex interplay between demographic shifts and evolving disease patterns, emphasizing that both changing population structures and ongoing epidemiologic dynamics shape the growing burden of asbestos-related cancers in China.

## Discussion

This study provides a comprehensive evaluation of the long-term burden of cancer attributable to occupational asbestos exposure in China from 1990 to 2021, revealing persistent and rising public health risks. In 2021, an estimated 29,020 deaths and more than 535,000 DALYs were linked to asbestos exposure, with the burden concentrated among older men. Lung cancer and mesothelioma accounted for most of the burden, followed by laryngeal, ovarian, and other cancers. The absolute number of asbestos-related cases increased steadily over three decades, largely due to population aging and growth. Age standardized rates showed a nonlinear pattern, rising sharply in the early 2000s, declining after 2011, and stabilizing or slightly increasing in recent years, particularly among women. Joinpoint and age period cohort analyses showed marked increases in the early 21st century and sustained burden in older age groups and early birth cohorts, consistent with long latency. Decomposition analysis indicated that deaths were mainly driven by aging and epidemiologic change, while DALYs were driven by changes in disease risk and population expansion. These findings highlight the substantial historical burden of asbestos exposure and its growing impact in aging populations in the absence of a national ban, underscoring the need for targeted occupational health interventions in China.

Asbestos carcinogenicity is well established and reflects multiple interrelated mechanisms. Amphibole and chrysotile fibers persist in lung and pleural tissues for decades, provoking chronic inflammation, genotoxic stress, and oxidative damage (23–25). After inhalation, fibers evade macrophage clearance and accumulate in respiratory epithelium, generating reactive oxygen and nitrogen species that cause DNA strand breaks, chromosomal aberrations, and point mutations (26–29). Indirect oncogenic pathways arise through cytokines such as TNF-*α* and IL-1β that promote proliferation, angiogenesis, and apoptosis resistance (30, 31). Fibers also disrupt mitotic spindle function and cell cycle control, increasing malignant transformation (32, 33). Mesothelioma is closely linked to chronic mesothelial irritation and iron-rich asbestos bodies that amplify mutagenic oxidative reactions (34). These mechanisms align with the long latency observed, with peak burden in those aged 70 years and older. Systemic fiber distribution via lymphatic or circulatory routes may underlie extra-pulmonary associations, including ovarian and laryngeal cancers (35, 36).

Several characteristic trends emerged, particularly regarding sex differences, drivers of change, and generational exposure patterns. Males consistently bore a substantially higher asbestos-related cancer burden than females across all indicators, largely reflecting historical occupational segregation; in China, asbestos-intensive sectors such as shipbuilding, construction, and insulation predominantly employed men, resulting in greater cumulative exposure (37). Although occupational exposure remains primary, recent years have shown notable increases in DALYs and YLDs among females, especially after 2017. This pattern should be interpreted cautiously; plausible contributors include rising female participation in industry, environmental or secondary exposure from contaminated household dust or cohabitation with exposed workers, and improved case ascertainment/diagnostic capacity that may enhance detection in women (38, 39). In addition, the sharp rise observed in the early 2000s likely reflects a combination of true risk elevation (consistent with latency and cohort effects) and better reporting/coding rather than incidence alone. Decomposition analysis indicated that mortality increases were mainly driven by population aging, whereas the rise in DALYs was chiefly linked to epidemiologic change and population growth. This divergence suggests that older populations are increasingly succumbing to asbestos-related cancers while ongoing exposure risks and demographic expansion generate new fatal and nonfatal cases. Age-period-cohort modeling showed sharp elevations in DALY and death rates among early birth cohorts before 1940, consistent with peak industrial asbestos use, with progressively lower burdens in later cohorts. Nonetheless, rising burden in older adults and the growing impact among females underscore needs for proactive surveillance, prevention across generations, and broadened, evidence-based occupational safety frameworks addressing nontraditional and gender-specific exposures.

The findings have important implications for clinical practice and occupational health policy in China. Clinically, the growing burden of asbestos-related malignancies, especially lung cancer and mesothelioma, warrants earlier detection focused on high-risk groups, particularly older men with documented exposure. Diagnostic delays drive late presentation and poor outcomes; routine screening for retired industrial workers and expanded access to low-dose computed tomography for early lung cancer detection could improve prognosis (40). From a policy perspective, the evidence supports a comprehensive national ban on all forms of asbestos, including chrysotile, which remains in use despite its carcinogenicity (41). A centralized registry of asbestos-exposed workers linked to hospital cancer databases should enable long-term monitoring, timely diagnosis, and fair compensation. Regular workplace inspections and exposure documentation are essential, particularly in legacy industries. International models offer guidance, including Italy’s National Mesothelioma Registry, which links occupational histories with diagnoses (42), and Australia’s Asbestos Safety and Eradication Agency, which coordinates risk management and public education (43). Rising DALYs and YLDs among women argue for gender-sensitive surveillance, while epidemiologic drivers highlight modifiable gaps in timely diagnosis and treatment access. Combined clinical and policy actions could curtail future burden.

Despite strengths in using GBD 2021 data and multiple analytic frameworks, this study has limitations. First, estimates rely on modeled data rather than individual-level exposure. The Asbestos Impact Ratio (AIR) is a validated proxy but may not capture China’s heterogeneous exposure landscape, including regional disparities in production and use, informal and undocumented sectors, and historical shifts in industry, leading to under or overestimation in subpopulations (44). In addition, all incidence and temporal trends derive from GBD 2021 rather than from linked national compensation or occupation-notified case series; GBD serves as a pragmatic proxy for China but cannot replace province-level exposure records or job-category detail. Second, long disease latency may cause current estimates to understate future incidence among groups exposed in recent decades, particularly in undocumented industries. Third, although the GBD study uses tools such as CODEm and redistribution algorithms to address misclassification and underreporting, constraints persist in resource-limited settings with low diagnostic capacity. Incomplete ICD specificity and misclassification of mesothelioma and other asbestos-related cancers may still affect accuracy (45, 46). We did not conduct misclassification-specific sensitivity analyses beyond the 95% uncertainty intervals reported by GBD, so some residual bias cannot be excluded. This analysis also does not account for co-exposures that may act synergistically with asbestos, such as silica dust or smoking (47). Moreover, we lack nationally representative data on (i) fiber mineralogy/chemistry (e.g., TEM/SEM-EDS confirmation of amphibole admixture in chrysotile), (ii) time-series on domestic/imported asbestos use by sector and province, and (iii) occupation-specific exposure information (e.g., mining, processing, insulation, shipbuilding); these gaps limit subnational attribution and causal inference. Future work should build regionally and sectorally detailed exposure databases, conduct longitudinal cohorts to validate modeled estimates, and apply molecular epidemiology to clarify gene environment interactions. Priority actions include establishing a centralized registry of asbestos-exposed workers linked to cancer registries, implementing routine compositional surveillance of mined/imported materials, and harmonizing provincial surveillance to enable latency-aware, occupation-specific analyses. Sex-specific behavioral and occupational factors, integration of hospital cancer registries, and strengthened surveillance will improve estimation and guide targeted prevention, compensation, and progress monitoring.

## Conclusion

Occupational asbestos exposure remains a critical public health concern, with long-term implications for cancer prevention and control. This study underscores the importance of sustained efforts to monitor and address asbestos-related disease burden within rapidly aging populations. Strengthening occupational health policies, expanding exposure surveillance, and promoting early cancer detection are essential steps toward mitigating future impact. There is also a pressing need for comprehensive regulatory measures, including the implementation of a national asbestos ban and targeted interventions for historically exposed cohorts. Future research should focus on integrating individual-level exposure data, exploring gene–environment interactions, and improving diagnostic precision for asbestos-related malignancies. Longitudinal studies and high-resolution modeling will be instrumental in refining burden estimates and informing evidence-based policy. Continued interdisciplinary collaboration between clinicians, public health authorities, and policymakers will be key to reducing the legacy of asbestos exposure and improving long-term cancer outcomes.

## Data Availability

Publicly available datasets were analyzed in this study. This data can be found here: https://vizhub.healthdata.org/gbd-results/.

## Supplementary material

Supplementary Figure S1 Comparison of crude number and rate of total cancer burden attributable to occupational asbestos exposure in China by age group in 1990 and 2021. **(A)** Number and crude mortality rate per 100,000 population. **(B)** Number and crude DALY rate per 100,000 population. **(C)** Number and crude YLD rate per 100,000 population. **(D)** Number and crude YLL rate per 100,000 population. DALYs, disability-adjusted life years; YLDs, years lived with disability; YLLs, years of life lost.

Supplementary Figure S2 Sex distribution of cancer burden attributable to occupational asbestos exposure by cancer type in China in 2021. **(A)** Sex distribution of deaths, **(B)** DALYs, **(C)** YLDs, and **(D)** YLLs for major asbestos-related cancers. Abbreviations: DALYs, disability-adjusted life years; YLDs, years lived with disability; YLLs, years of life lost.

Supplementary Figure S3 Age-period-cohort analysis of mortality rates for total cancer attributable to occupational asbestos exposure in China, 1990-2021. **(A)** The age-specific mortality rates according to time periods; each line connects the mortality rates for a given 5-year period. **(B)** The age-specific mortality rates according to birth cohorts; each line connects the mortality rates for a given 5-year birth cohort. **(C)** The period-specific mortality rates according to age groups; each line connects the mortality rates for a given 5-year age group. **(D)** The cohort-specific mortality rates according to age groups; each line connects the mortality rates for a given 5-year age group.
