# Supplementary figures and images for: Burden and trends of cancer attributable to occupational asbestos exposure in China from 1990 to 2021
Source: Front Public Health. 2026 Jan 5;13:1672598. doi: 10.3389/fpubh.2025.1672598 (PMC12813111; doi:10.3389/fpubh.2025.1672598)

A

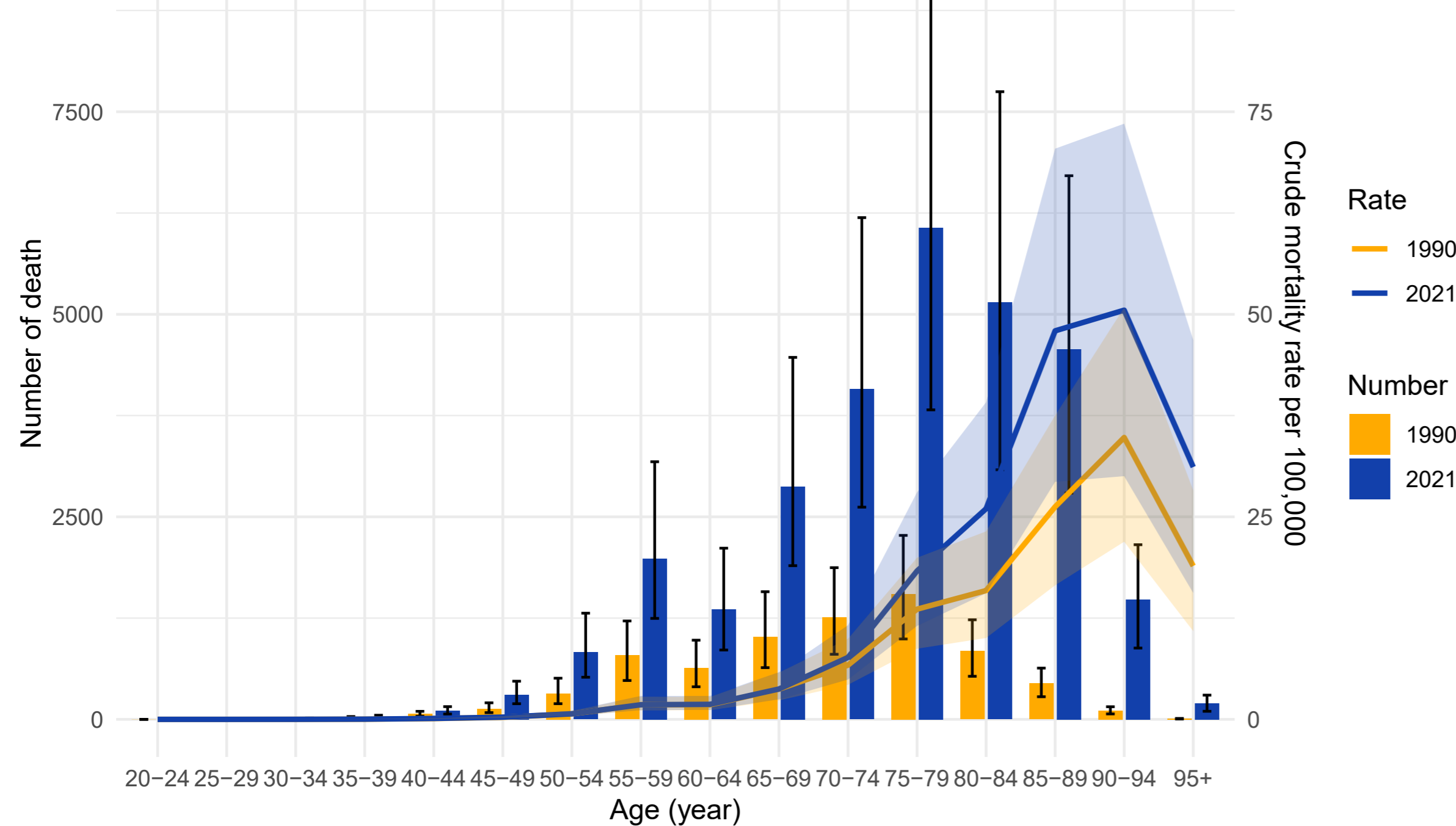

B

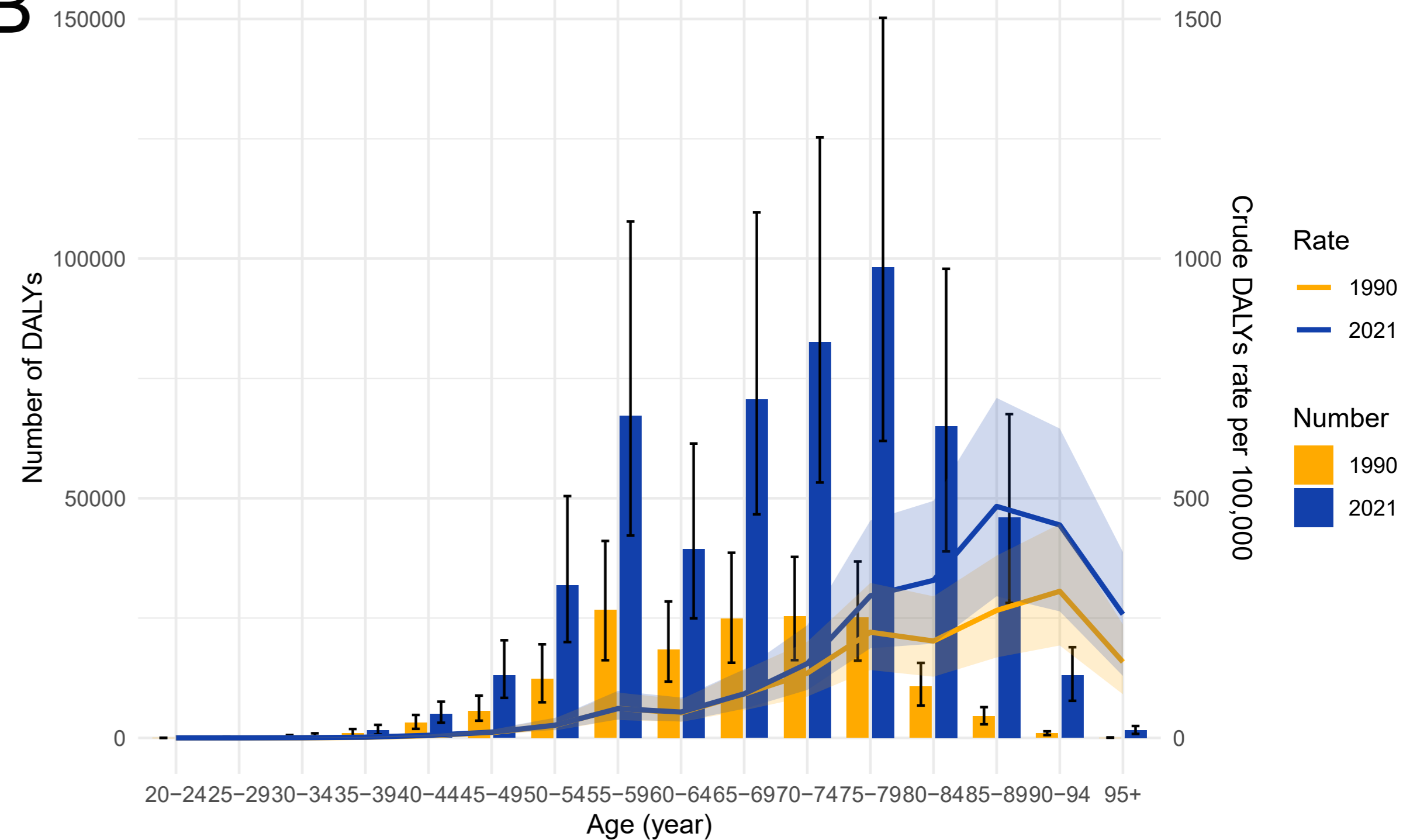

C

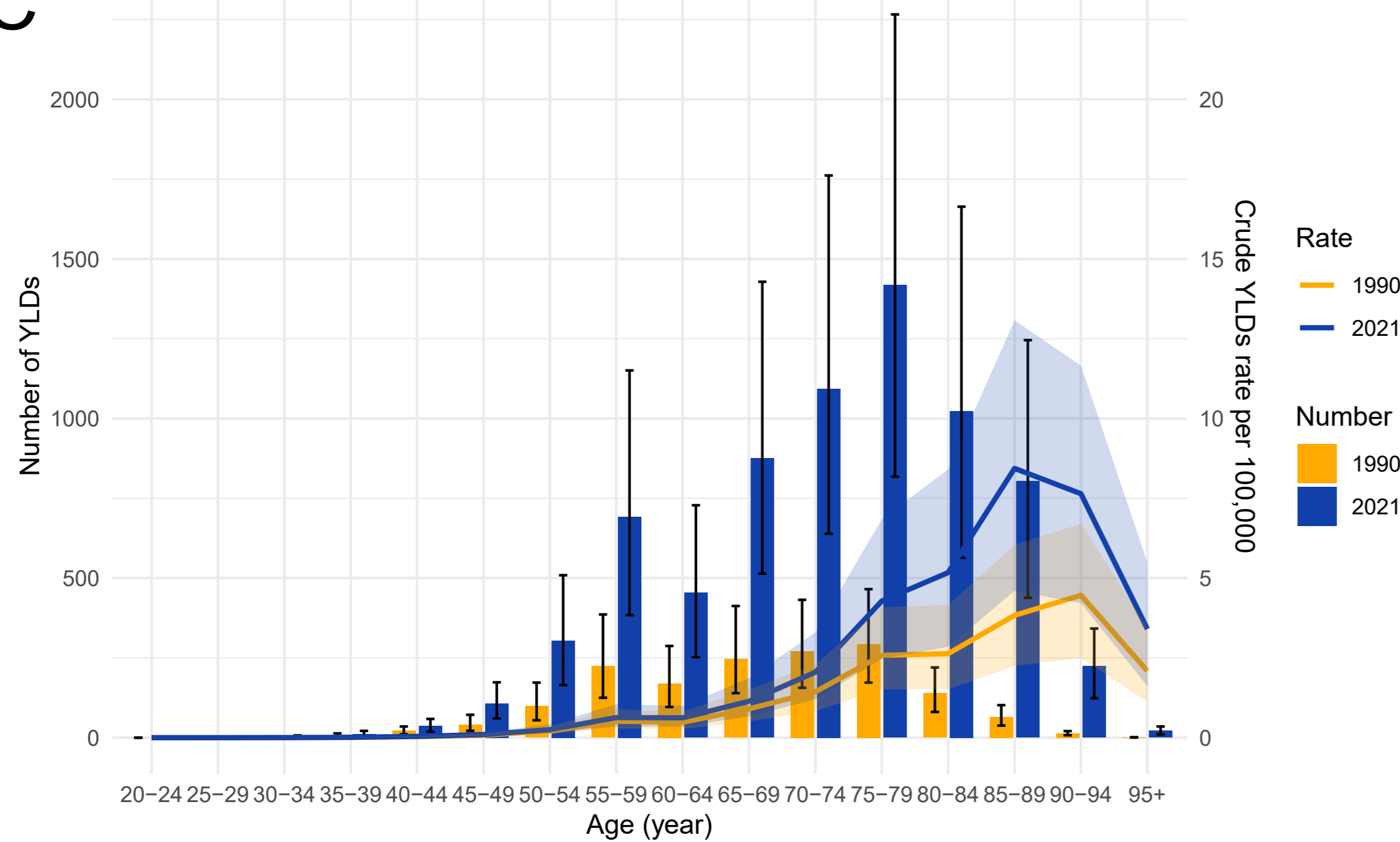

D

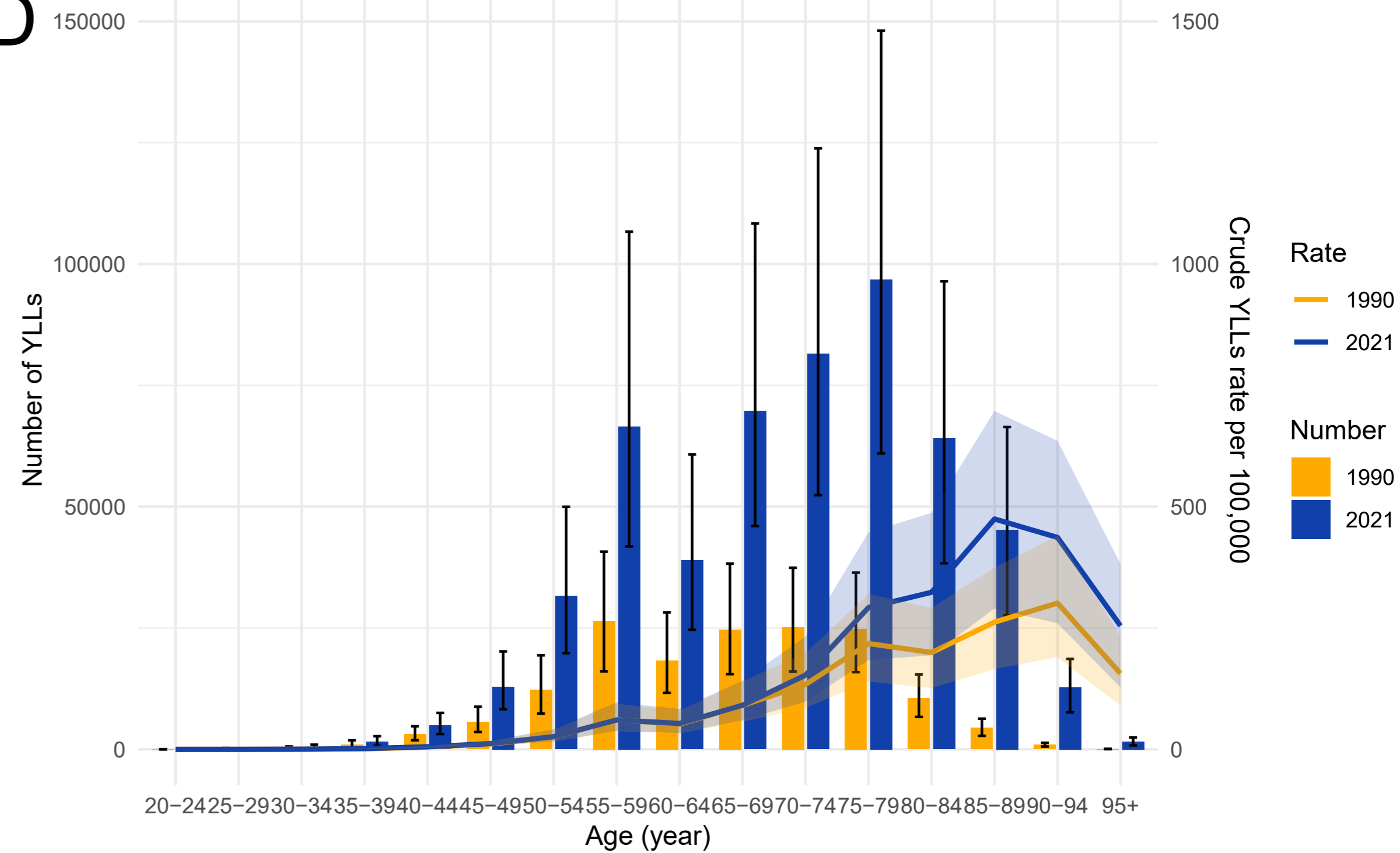

Supplement: Supplementary Figure S1 — Comparison of crude number and rate of total cancer burden attributable to occupational asbestos exposure in China by age group in 1990 and 2021. (A) Number and crude mortality rate per 100,000 population. (B) Number and crude DALY rate per 100,000 population. (C) Number and crude YLD rate per 100,000 population. (D) Number and crude YLL rate per 100,000 population. DALYs, disability-adjusted life years; YLDs, years lived with disability; YLLs, years of life lost. [file Data_Sheet_1.PDF]

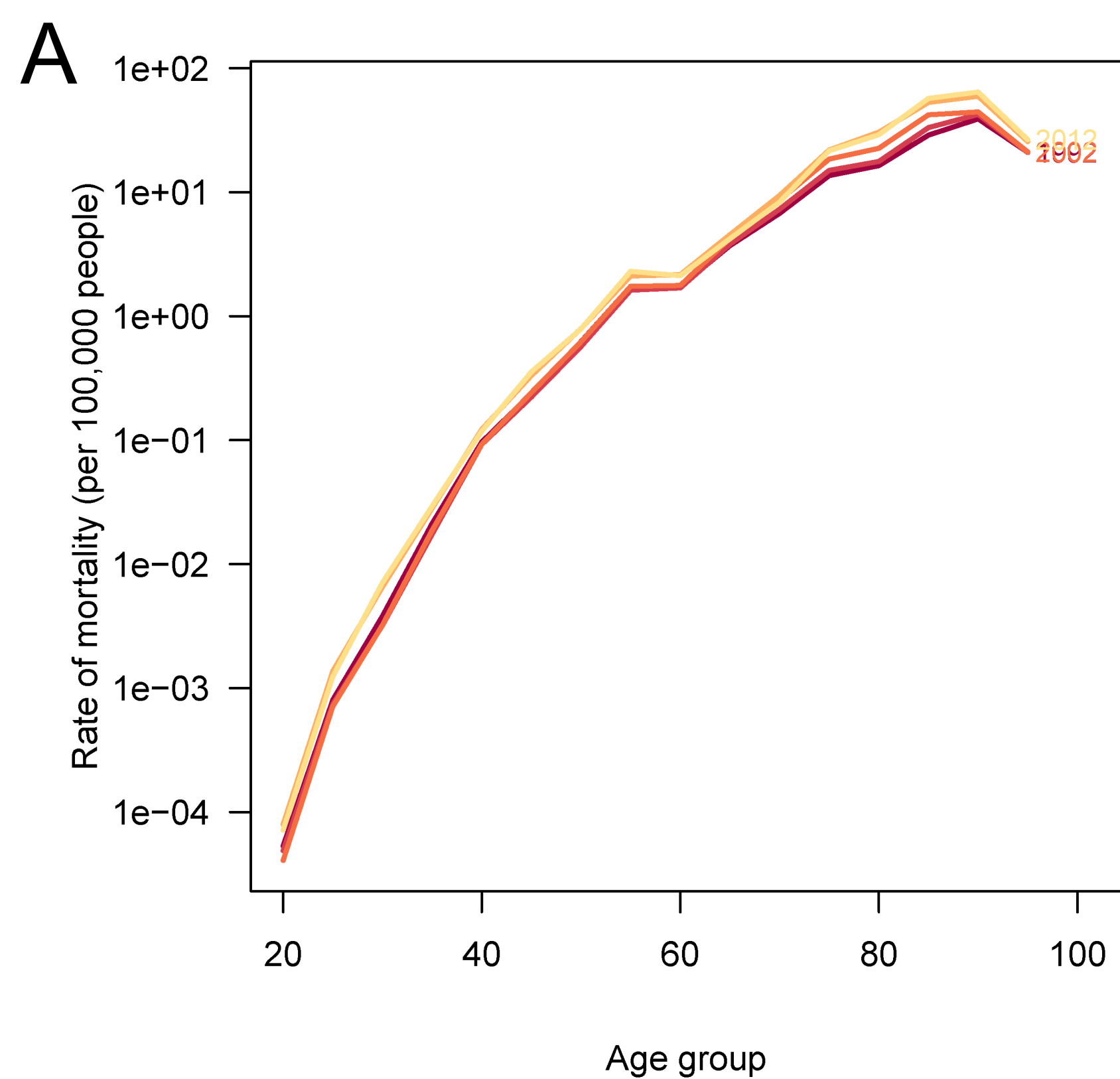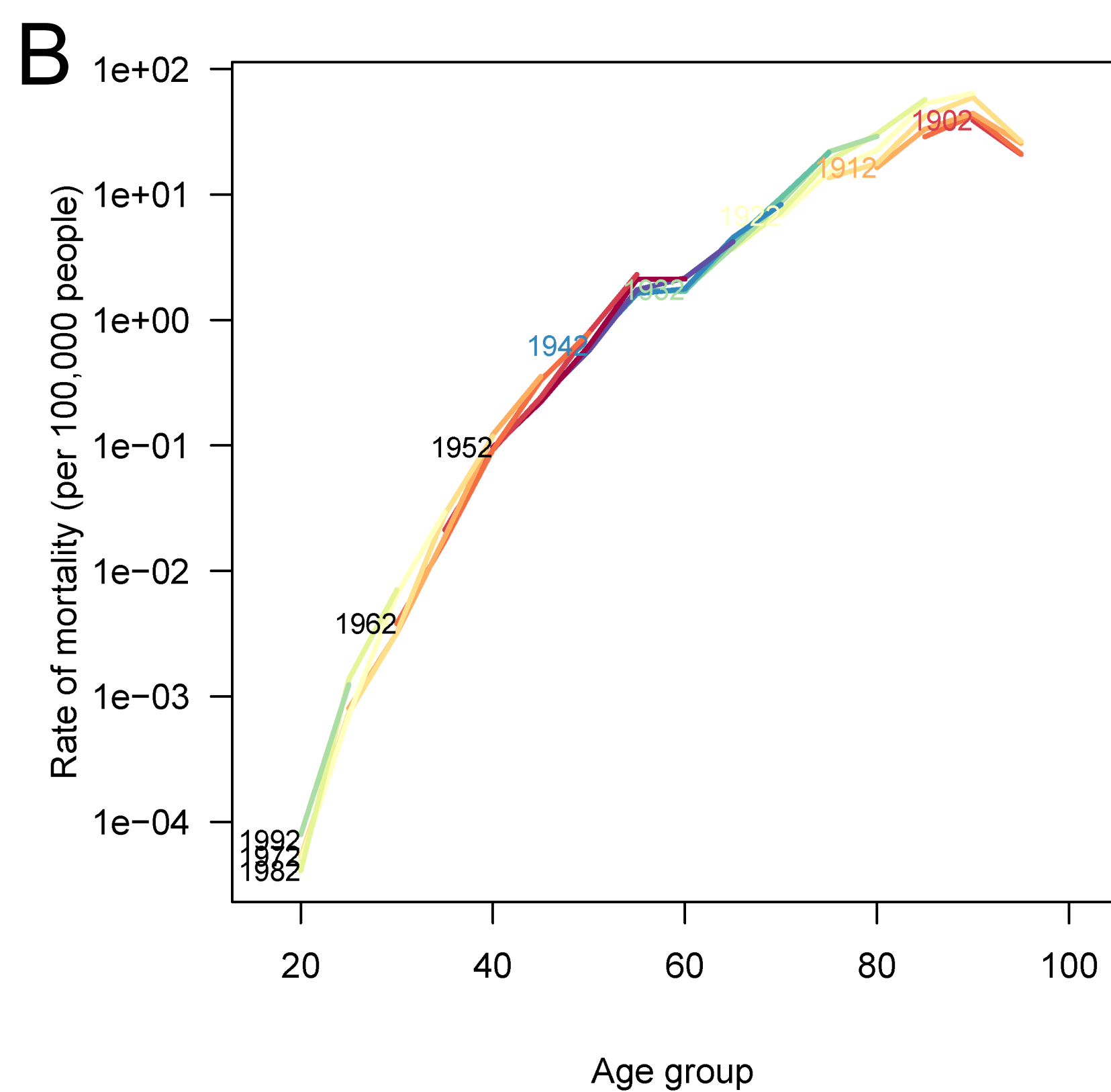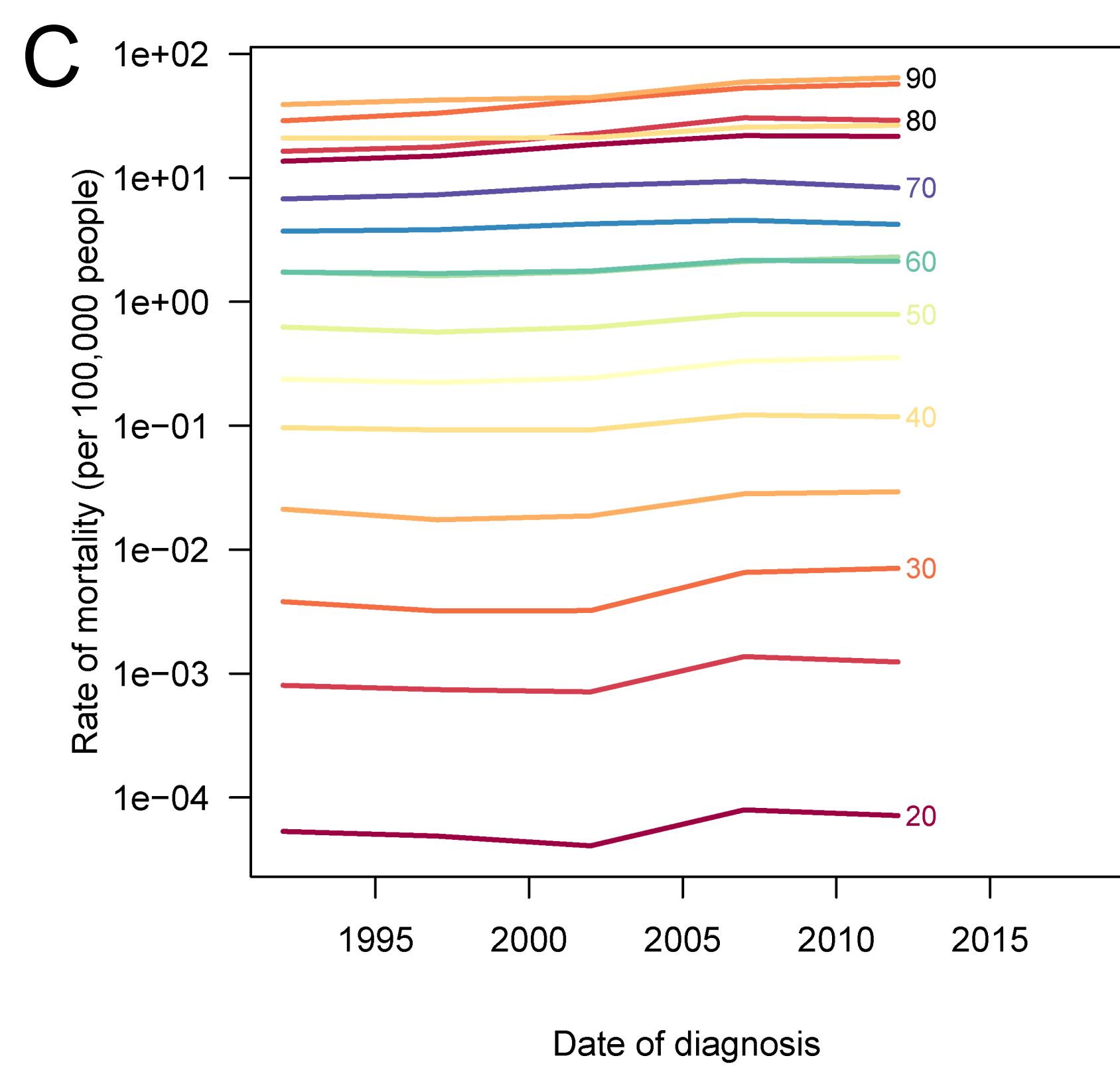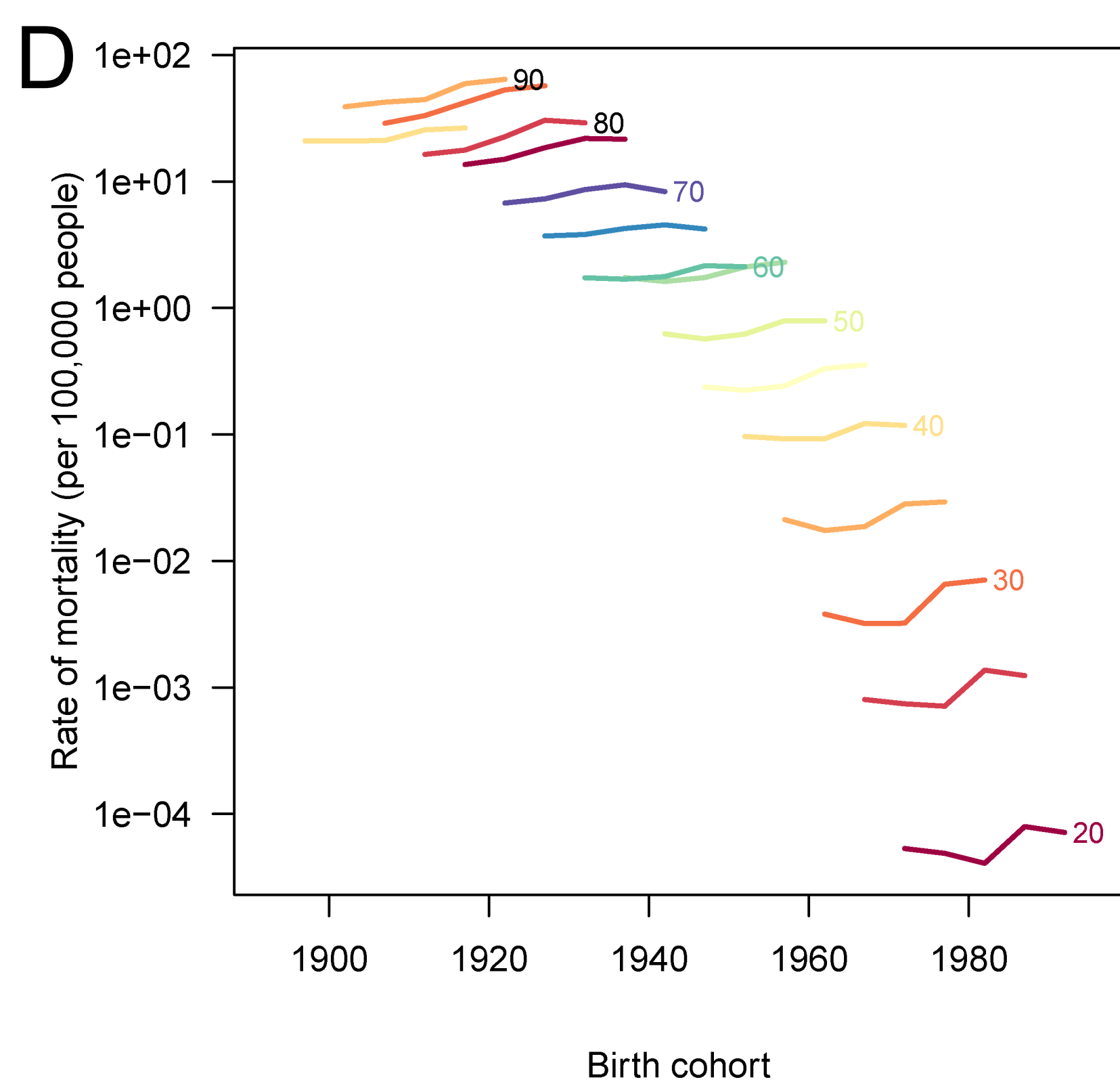

Supplement: Supplementary Figure S3 — Age-period-cohort analysis of mortality rates for total cancer attributable to occupational asbestos exposure in China, 1990-2021. (A) The age-specific mortality rates according to time periods; each line connects the mortality rates for a given 5-year period. (B) The age-specific mortality rates according to birth cohorts; each line connects the mortality rates for a given 5-year birth cohort. (C) The period-specific mortality rates according to age groups; each line connects the mortality rates for a given 5-year age group. (D) The cohort-specific mortality rates according to age groups; each line connects the mortality rates for a given 5-year age group. [file Data_Sheet_3.PDF]
