# Supplementary material for: Burden and trends of cancer attributable to occupational asbestos exposure in China from 1990 to 2021
Source: Front Public Health. 2026 Jan 5;13:1672598. doi: 10.3389/fpubh.2025.1672598 (PMC12813111; doi:10.3389/fpubh.2025.1672598)
Supplement: Supplementary Figure S2 — Sex distribution of cancer burden attributable to occupational asbestos exposure by cancer type in China in 2021. (A) Sex distribution of deaths, (B) DALYs, (C) YLDs, and (D) YLLs for major asbestos-related cancers. Abbreviations: DALYs, disability-adjusted life years; YLDs, years lived with disability; YLLs, years of life lost. [file Data_Sheet_2.PDF]

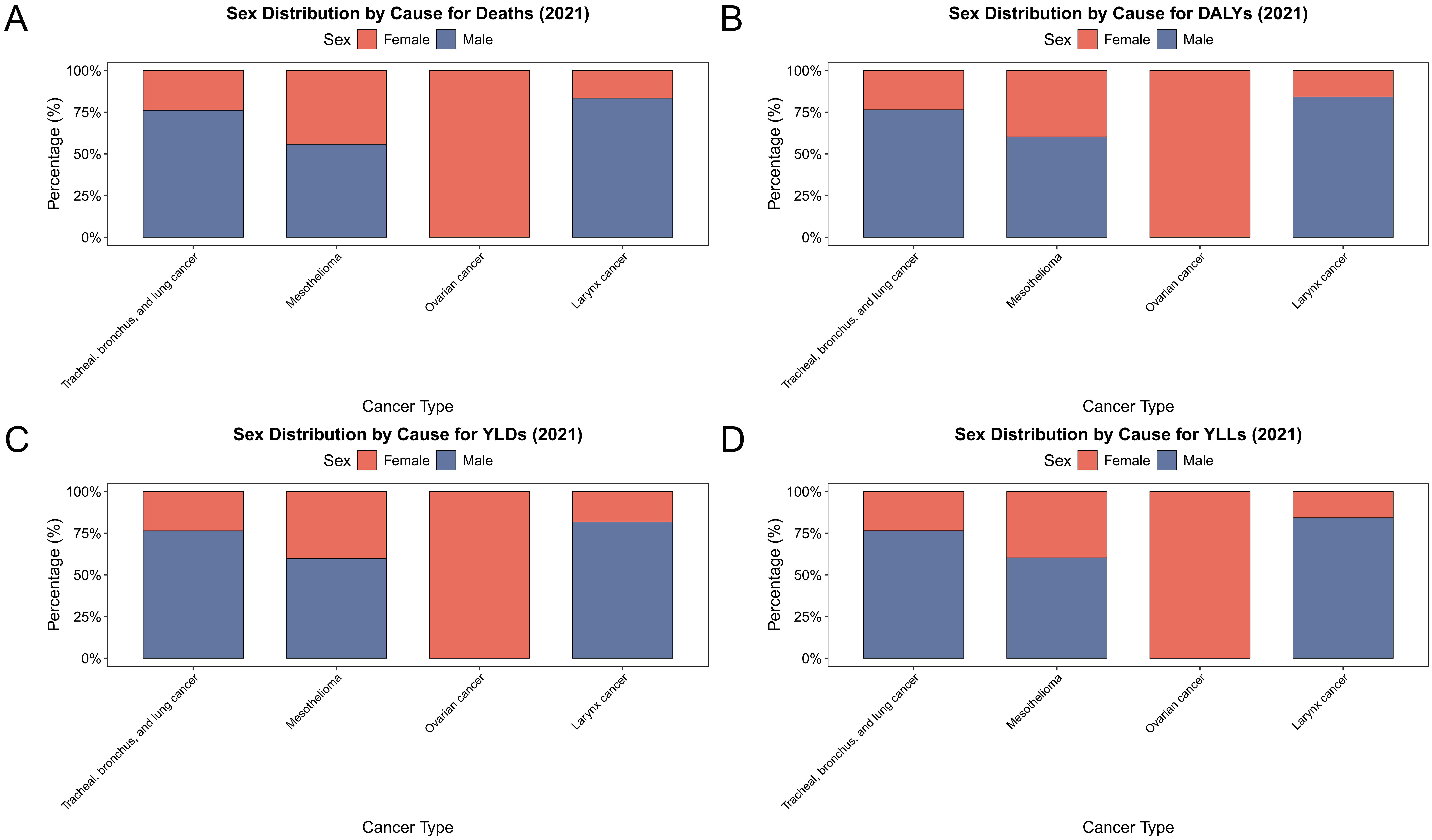
A
Sex Distribution by Cause for Deaths (2021)
B
Sex Distribution by Cause for DALYs (2021)
C
Sex Distribution by Cause for YLDs (2021)
D
Sex Distribution by Cause for YLLs (2021)
Sex
Female
Male
Percentage (%)
100%
75%
50%
25%
0%
Tracheal, bronchus, and lung cancer
Mesothelioma
Ovarian cancer
Larynx cancer
Cancer Type
