# Supplementary material for: Burden and trends of cancer attributable to occupational asbestos exposure in China from 1990 to 2021
Source: Front Public Health. 2026 Jan 5;13:1672598. doi: 10.3389/fpubh.2025.1672598 (PMC12813111; doi:10.3389/fpubh.2025.1672598)
Supplement: Supplementary file 4 [file Table_1.DOCX]

Supplementary Table S1. YLDs and YLLs for total cancer attributable to occupational asbestos exposure in China, 2021, with trends in ASRs per 100,000 population, 1990 - 2021

|  | YLDs | | | YLLs | | |
| --- | --- | --- | --- | --- | --- | --- |
| Cancer type | No, in thousands | Age-standardized rate per 100,000 | Percentage change from 1990 to 2021 | No, in thousands | Age-standardized rate per 100,000 | Percentage change from 1990 to 2021 |
| Total cancers | 7.1 (4.1, 11.2) | 0.3 (0.2, 0.6) | 56.9 (13.6, 117.7) | 528.7 (342.2, 792.8) | 25.5 (16.5, 37.9) | 23.6 (-12.5, 74.2) |
| Tracheal, bronchus, and lung cancer | 6 (3.3, 9.8) | 0.3 (0.2, 0.5) | 67.6 (18.2, 138.4) | 454.9 (275.2, 702.9) | 22.1 (13.3, 33.9) | 26.8 (-11.6, 83.6) |
| Mesothelioma | 0.7 (0.4, 1) | 0 (0, 0) | 12.6 (-21.4, 55.3) | 62.1 (45.9, 80.4) | 2.9 (2.1, 3.7) | 11.6 (-26.3, 58.1) |
| Ovarian cancer | 0.2 (0.1, 0.4) | 0 (0, 0) | 14.9 (-38.1, 100.2) | 7.4 (2.5, 14.3) | 0.3 (0.1, 0.7) | -3.6 (-47.1, 66) |
| Larynx cancer | 0.2 (0.1, 0.3) | 0 (0, 0) | 24.9 (-15.4, 88) | 4.2 (2.2, 7) | 0.2 (0.1, 0.3) | -30.7 (-54, 4.6) |

Values in parentheses indicate 95% UIs, estimated using Monte Carlo simulations. Abbreviations: YLDs, years lived with disability; YLLs, years of life lost; ASRs, age-standardized rates; UI, uncertainty interval.
