# Supplementary material for: Burden and trends of cancer attributable to occupational asbestos exposure in China from 1990 to 2021
Source: Front Public Health. 2026 Jan 5;13:1672598. doi: 10.3389/fpubh.2025.1672598 (PMC12813111; doi:10.3389/fpubh.2025.1672598)
Supplement: Supplementary file 5 [file Table_2.DOCX]

Supplementary Table S2. Trends in age-standardized mortality, DALY, YLD, and YLL rates (per 100,000 persons) among both sexes, males, and females from 1990 to 2021 for total cancer attributable to occupational asbestos exposure in China.

|  | Age-standardized mortality rate | | | Age-standardized DALY rate | | | Age-standardized YLD rate | | | Age-standardized YLL rate | | |
| --- | --- | --- | --- | --- | --- | --- | --- | --- | --- | --- | --- | --- |
| Gender | Period | APC (95% CI) | AAPC (95% CI) | Period | APC (95% CI) | AAPC (95% CI) | Period | APC (95% CI) | AAPC (95% CI) | Period | APC (95% CI) | AAPC (95% CI) |
| Both | 1990-1998 | 0.61 (0.17 - 0.94) ^*^ | 1.00 (0.90 - 1.08) ^*^ | 1990-1998 | 0.04 (-0.40 - 0.34) | 0.71 (0.62 - 0.78) ^*^ | 1990-1998 | 0.48 (0.07 - 0.76) ^*^ | 1.46 (1.37 - 1.52) ^*^ | 1990-1998 | 0.03 (-0.40 - 0.34) | 0.70 (0.61 - 0.77) ^*^ |
|  | 1998-2007 | 3.41 (2.93 - 3.72) ^*^ |  | 1998-2007 | 3.07 (2.62 - 3.38) ^*^ |  | 1998-2007 | 3.90 (3.56 - 4.20) ^*^ |  | 1998-2007 | 3.06 (2.61 - 3.36) ^*^ |  |
|  | 2007-2011 | 5.40 (4.70 - 6.34) ^*^ |  | 2007-2011 | 5.03 (4.40 - 5.93) ^*^ |  | 2007-2011 | 6.42 (5.79 - 7.44) ^*^ |  | 2007-2011 | 5.02 (4.39 - 5.91) ^*^ |  |
|  | 2011-2018 | -3.50 (-4.03 - -3.19) ^*^ |  | 2011-2018 | -3.49 (-4.00 - -3.19) ^*^ |  | 2011-2017 | -2.91 (-3.58 - -2.54) ^*^ |  | 2011-2018 | -3.50 (-4.00 - -3.20) ^*^ |  |
|  | 2018-2021 | -0.09 (-1.43 - 1.98) |  | 2018-2021 | -0.12 (-1.43 - 1.92) |  | 2017-2021 | -0.16 (-1.04 - 1.51) |  | 2018-2021 | -0.13 (-1.42 - 1.91) |  |
| Female | 1990-1998 | 0.30 (-0.14 - 0.60) | 1.03 (0.95 - 1.09) ^*^ | 1990-1998 | -0.02 (-0.41 - 0.28) | 0.81 (0.73 - 0.87) ^*^ | 1990-1999 | 0.48 (0.17 - 0.71) ^*^ | 1.47 (1.40 - 1.53) ^*^ | 1990-1998 | -0.02 (-0.41 - 0.27) | 0.80 (0.72 - 0.86) ^*^ |
|  | 1998-2007 | 2.22 (1.88 - 2.56) ^*^ |  | 1998-2007 | 2.07 (1.74 - 2.37) ^*^ |  | 1999-2007 | 2.92 (2.47 - 3.28) ^*^ |  | 1998-2007 | 2.06 (1.73 - 2.36) ^*^ |  |
|  | 2007-2011 | 5.50 (4.88 - 6.58) ^*^ |  | 2007-2011 | 4.69 (4.12 - 5.60) ^*^ |  | 2007-2011 | 5.88 (5.28 - 6.91) ^*^ |  | 2007-2011 | 4.67 (4.10 - 5.58) ^*^ |  |
|  | 2011-2015 | -4.05 (-4.96 - -3.48) ^*^ |  | 2011-2016 | -3.35 (-3.93 - -2.95) ^*^ |  | 2011-2016 | -2.67 (-3.34 - -2.27) ^*^ |  | 2011-2016 | -3.36 (-3.94 - -2.96) ^*^ |  |
|  | 2015-2021 | 0.78 (0.33 - 1.32) ^*^ |  | 2016-2021 | 1.07 (0.50 - 1.87) ^*^ |  | 2016-2021 | 1.72 (1.18 - 2.54) ^*^ |  | 2016-2021 | 1.07 (0.49 - 1.85) ^*^ |  |
| Male | 1990-1998 | 0.66 (-0.08 - 1.19) | 0.89 (0.77 - 1.00) ^*^ | 1990-1998 | 0.05 (-0.44 - 0.39) | 0.64 (0.54 - 0.72) ^*^ | 1990-1998 | 0.52 (-0.01 - 0.88) | 1.43 (1.32 - 1.51) ^*^ | 1990-1998 | 0.05 (-0.45 - 0.38) | 0.63 (0.53 - 0.71) ^*^ |
|  | 1998-2007 | 3.94 (0.71 - 4.30) ^*^ |  | 1998-2007 | 3.51 (2.67 - 3.83) ^*^ |  | 1998-2007 | 4.44 (3.73 - 4.81) ^*^ |  | 1998-2007 | 3.50 (2.65 - 3.81) ^*^ |  |
|  | 2007-2011 | 5.13 (-3.00 - 6.12) |  | 2007-2011 | 5.01 (4.17 - 5.90) ^*^ |  | 2007-2011 | 6.26 (5.39 - 7.26) ^*^ |  | 2007-2011 | 4.99 (4.16 - 5.89) ^*^ |  |
|  | 2011-2018 | -4.07 (-5.14 - -3.66) ^*^ |  | 2011-2018 | -3.94 (-4.62 - -3.61) ^*^ |  | 2011-2018 | -3.20 (-3.96 - -2.84) ^*^ |  | 2011-2018 | -3.95 (-4.62 - -3.61) ^*^ |  |
|  | 2018-2021 | -1.13 (-2.70 - 1.15) |  | 2018-2021 | -1.03 (-2.44 - 0.97) |  | 2018-2021 | -0.30 (-1.83 - 1.74) |  | 2018-2021 | -1.04 (-2.45 - 0.97) |  |

Abbreviations: DALYs, disability-adjusted life-years; YLDs, years lived with disability; YLLs, years of life lost; AAPC, average annual percent change presented for full period; APC, annual percent change; CI, confidence interval. ^*^, *p* <0.05.
